# INSIGHTS INTO HEALTH-RELEVANT INTERPERSONAL DYNAMICS IN AGING COUPLES FROM THE LAB AND DAILY LIFE

**DOI:** 10.1093/geroni/igac059.729

**Published:** 2022-12-20

**Authors:** Theresa Pauly, Karolina Kolodziejczak, Christina Röcke

## Abstract

Emerging research demonstrates that long-term and daily health indicators are closely linked in aging partners. Yet, not much is known about exactly how partners get under each other’s skin. This symposium investigates different positive and negative interpersonal contexts, including physical intimacy, positive and negative emotional experiences, and couple conversations (e.g., conflict, recounting distressing memories, discussing enjoyable topics), and their link with neuroendocrine and cardiovascular markers. The four talks feature a variety of study designs ranging from laboratory research to ambulatory assessment methods which recruited couples in midlife and old age. Kolodziejczak et al. use ambulatory assessment data to examine links between (experienced and wished for) physical intimacy, affect, and cortisol in older couples. Pauly et al. pool data from three ambulatory assessment aging studies to analyze how own and partner positive and negative affective states are intertwined with everyday cortisol secretion in old age. Wilson et al. invited middle-aged to older couples to engage in two different conversations (recounting a difficult memory, conflict) and investigated concurrent changes in affect and cardiovascular activity. Meier and colleagues make use of automated language analysis to examine how positive and negative emotion word use during a positive and negative conversation in the laboratory relates to cardiovascular reactivity in middle-aged couples. The discussion by Christina Röcke will delineate insights gained from these four papers, discuss their strengths and limitations, and outline directions for future inquiry.

